# Relationship between visit-to-visit blood pressure variability and depressive mood in Korean primary care patients

**DOI:** 10.1186/s12875-024-02404-6

**Published:** 2024-05-07

**Authors:** Ga Hee Lee, Jung Ah Lee, Sung Sunwoo, Young Sik Kim

**Affiliations:** 1 Department of Family Medicine, Asan Medical Center, University of Ulsan College of Medicine, 88 Olympic-Ro 43-Gil, Songpa-gu, Seoul, 05505 South Korea; 2 Department of Family Medicine, Korea University Ansan Hospital, Korea University College of Medicine, Asan, Gyeonggi-do South Korea

**Keywords:** Depressive mood, Blood pressure, Blood pressure variability, Primary care, Visit-to-visit blood pressure variability

## Abstract

**Background:**

High blood pressure variability (BPV) increases the risk of cardiovascular disease and may be better prognostic factor than blood pressure. Depressive mood is a common symptom among patients visiting primary care. This study aimed to investigate the association between depressive mood and high BPV among Korean primary care patients.

**Methods:**

The Family Cohort Study in Primary Care (FACTS), conducted from April 2009 to November 2017, utilized a prospective cohort of Korean primary care patients, with a median follow-up period of 7.25 years. Depressive mood was assessed as a score of 21 points or more on the Korean-type Center for Epidemiologic Studies Depression scale. BP was measured at the initial visit and first and second follow-up visit. Visit-to visit SBP variability was analyzed using four metrics: intra-individual standard deviation, coefficient of variation, variation independent of mean, and average real variability. Logistic regression analysis was used to estimate the association of high BPV with depressive mood and other variables.

**Results:**

Among 371 participants, 43 (11.6%) had depressive mood based on depression scores. Older age (odds ratio [OR]: 1.04, 95% confidence interval [CI]: 1.01–1.07) were associated with high SBP variability regardless of taking antihypertensive medication. Among participants taking antihypertensive medication, those with depressive mood had twice the risk of high SBP variability compared with those who did not (OR: 2.95, 95% CI: 1.06–8.20).

**Conclusions:**

Depressive mood was associated with high visit-to-visit SBP variability in primary care patients taking antihypertensive medication, potentially indicating increased cardiovascular risk. Primary care physicians should therefore closely monitor BPV in patients with depressive symptoms and provide appropriate interventions.

## Introduction

Major depression is a prevalent condition that is frequently characterized by a chronic-recurrent course [1]. It affects approximately 2–4% of the general population and 10% of primary care patients [2]. Major depressive disorder stands as a leading cause of global disease burden [1, 3], with depressive symptoms being associated with major chronic and cardiovascular diseases [4–6].

Blood pressure variability (BPV) pertains to the fluctuations in blood pressure (BP) occurring within a specified timeframe, such as minutes, over a period of 24 h, or longer. This phenomenon is thought to result from intricate interactions involving extrinsic behavioral factors and intrinsic cardiovascular regulatory mechanisms [7]. Recent research indicates that BPV is independently associated with cardiovascular events and target organ damage [8, 9]. The 24-hour ambulatory BP monitoring method is commonly used to evaluate short-term BPV [10, 11], whereas long-term BPV is typically evaluated based on BP measurements obtained during periodic visits to clinics, commonly conducted monthly or yearly [11].

Previous studies have suggested that high BPV is associated with an elevated incidence of cardiovascular events [12], heightened cardiovascular risk, and increased mortality [13]. Although a definitive threshold for BPV elevating this risk remains undetermined, studies utilizing quartiles of standard deviation (SD) of BPV [13] and those based on the median value of SD of BPV [12] have consistently indicated that higher values are associated with an augmented risk of cardiovascular events. Additionally, another study has revealed that heightened BPV is associated with poor outcomes in cerebrovascular diseases [14]. This particularly study highlighted that elevated BPV, measured by the average absolute real variability (ARV) of BPV, serves as a predictive factor for poor short-term outcomes in patients with minor ischemic stroke.

The relationship between BPV and emotional status, particularly regarding depressive symptoms or anxiety, has been consistently observed in the literature [15–18]; however, few studies evaluated BPV as a factor. In the context of elderly-onset depression, evidence suggests an impact on diurnal variations in BP and an association with cerebral infarction [16]. Furthermore, a study reported a significant association between late-onset depression and higher systolic BPV [17]. Despite the established correlation between depression and BPV, there is a paucity of research on the association between long-term visit-to-visit BPV and depression [15]. Therefore, this study aimed to evaluate the influence of depressive mood on long-term visit-to-visit BPV among primary care patients in Korea.

## Methods

### Study participants

The Family Cohort Study in Primary Care (FACTS) was established to evaluate the effects of the familial environment on the health of primary care patients. The study cohort comprised couples and included married, cohabitating, separated, and divorced individuals. Both partners of the couples were recruited among individuals aged between 40 and 75 years who sought primary care physicians for periodic health checkups or treatment of chronic diseases such as hypertension, diabetes, and dyslipidemia. Follow-up began at the first visit to the Department of Family Medicine at one of 22 university hospitals nationwide from April 2009 to June 2011. The final date of follow-up was November 2017. The median follow-up period was 7.25 years. All participants provided written informed consent, and the survey received approval from the Institutional Review Board of Asan Medical Center (2016 − 1183).

### Demographic characteristics of study participants

Demographic characteristics were prospectively collected by interviewers or primary care physicians using questions regarding education status, monthly income, and medical history, including hypertension, diabetes, and hyperlipidemia. Educational level was categorized into three groups: < 12 years, 12 years, and > 12 years. Monthly income was evaluated by total household income using a single question and stratified into four categories: < 2.00 million Won ($1715), 2.00–3.99 million Won ($1715–3430), 4.00–5.99 million Won ($3430–5145), and ≥ 6.00 million Won ($5145).

The presence of hypertension, diabetes, or dyslipidemia was determined from the medical records of the study participants, identifying instances when the participants were reported to have any of these diseases and when they started taking antihypertensive medications, oral hypoglycemic agents, insulin, or lipid-lowering agents. Height and body weight were measured to the nearest 0.1 cm and 0.1 kg by trained interviewers. Body mass index (BMI) was calculated as (weight [kg])/(height [m])^2^ and categorized into three groups: < 23.0 kg/m^2^, 23.0–24.9 kg/m^2^, and ≥ 25.0 kg/m^2^. BP was measured from the left and right upper arm using a mercury manometer after a 10-minute resting period in a seated position [19]. These measurements were recorded as average BP for each visit.

### Definition of depressive mood and high visit-to-visit BPV

Depressive mood was assessed using the Korean-type Center for Epidemiologic Studies Depression (CES-D) scale, with a score of 21 points or more indicating the presence of depressive mood [20]. BP was measured at the initial visit and first and second follow-up visits, with follow-up intervals ranging between 6 and 24 months. Four metrics were used to assess the visit-to visit SBP variability: intra-individual SD, coefficient of variation (CV), variation independent of mean (VIM), and ARV as indices of visit-to visit SBP variability [21]. Among them, ARV was chosen for the primary analysis due to its comprehensive representation of visit-to-visit BPV. High visit-to-visit BPV was defined according to a previous study [14], noting elevated BPV as values higher than average ARV.

### Statistical analysis

Variables were presented as numbers with percentages or means with standard deviations (SDs). To compare characteristics between participants with and without depressive mood, the chi-square test was performed for categorical variables and Student’s *t*-test was performed for continuous variables. Additionally, the comparison of four metrics for evaluating visit-to-visit BPV of SBP included intra-individual SD, CV, VIM, and ARV. High BPV was defined when an individual’s ARV values exceeded the average ARV value of all participants. Binary logistic regression analysis was performed to estimate odds ratios (ORs) and 95% confidence intervals (CIs) for associations between high BPV and each variable, including depressive mood. Considering the potential influence of hypertension and hypertensive medication on BPV, the multivariable logistic analysis was conducted adjusting for these variables. Multivariable logistic regression analysis was performed to determine associations of high BPV with age, sex, BMI, and depressive mood. All analyses were performed using STATA version 18.0 (StataCorp, College Station, TX, USA) and SPSS ver. 21.0 (IBM Co., Armonk, NY, USA). A two-tailed P-value of < 0.05 was considered statistically significant.

## Results

### Characteristics of the participants

**Fig. 1:**
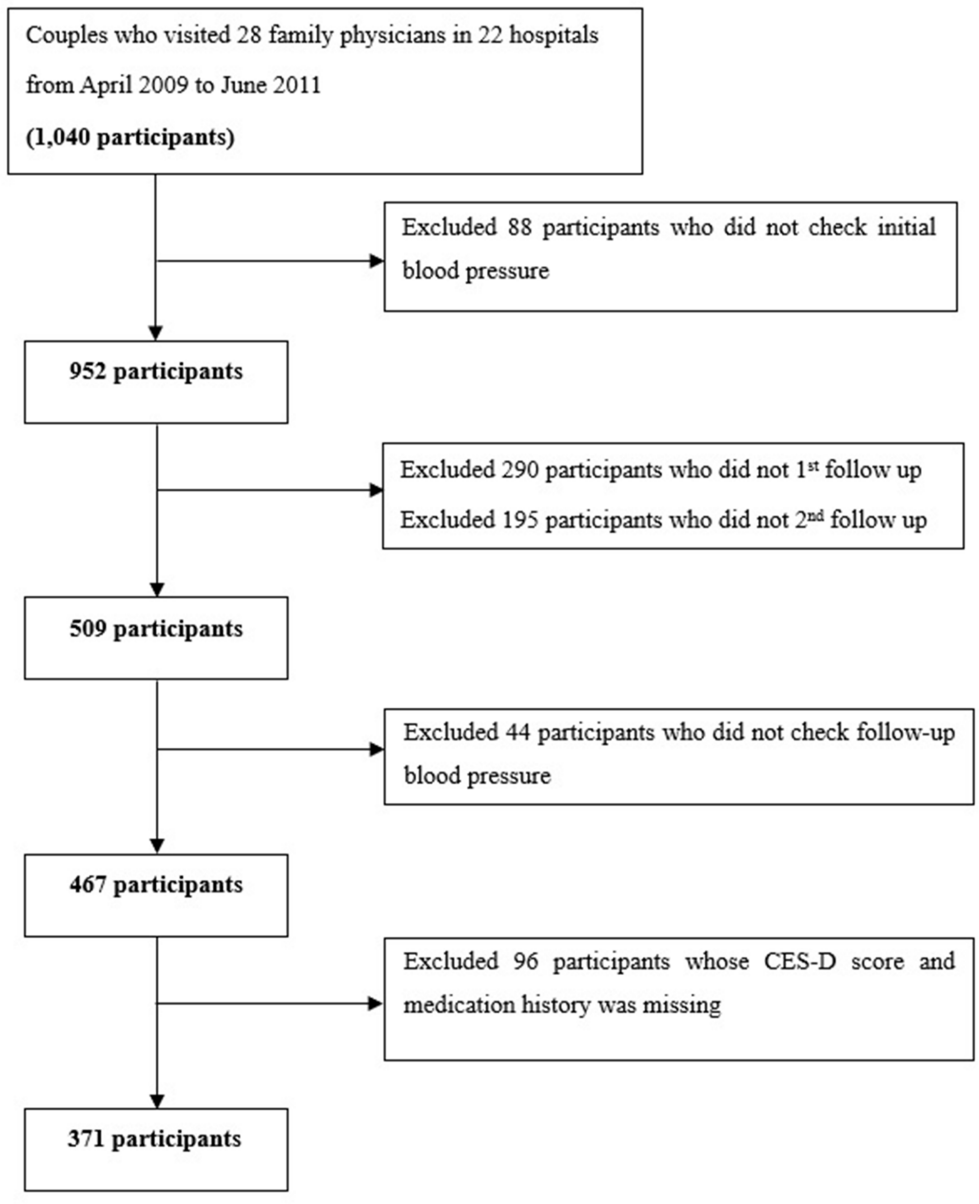
Flowchart of study participants

A total of 1040 participants were initially enrolled; however, 88 were excluded due to a lack of initial BP measurement. Among the remaining 952 participants, 485 were lost to first or second follow-up, 44 were excluded for not undergoing follow-up BP checks, and 52 were excluded due to missing CES-D scores or medical history (Fig. 1). Among the remaining 371 participants, 43 (11.6%) had depressive mood according to their CES-D scores. The baseline characteristics of these participants are shown in Table 1. The overall mean age was 60.08 ± 8.06 years, with no significant difference between participants with and without depressive mood (58.98 ± 7.61 vs. 60.22 ± 8.12 years, *P* = 0.343). A higher proportion of women than men had depressive mood (16.1% vs. 7.0%, *P* = 0.009), and more than half of the participants (55.8%) were taking antihypertensive medication; 19.4% were taking an oral hypoglycemic agent or insulin, and 41.2% were taking lipid-lowering agents. There were no significant differences in the histories of medications for hypertension, diabetes, and dyslipidemia between participants with and without depressive mood.

**Table 1:** Baseline characteristics of study participants

Characteristics	Total (*n* = 371)	Participants without depressive mood (*n* = 328)	Participants with depressive mood (*n* = 43)	*P*-value
	N (%) or mean (SD)			
Age (years)				
Mean (SD)	60.08 (8.06)	60.22 (8.12)	58.98 (7.61)	0.343
< 50	34 (9.2)	31 (91.2)	3 (8.8)	0.503
50–59	122 (32.9)	103 (84.4)	19 (15.6)	
60–69	178 (48.0)	161 (90.4)	17 (9.6)	
≥ 70	37 (10.0)	33 (89.2)	4 (10.8)	
Sex				
Men	185 (49.9)	172 (93.0)	13 (7.0)	0.009
Women	186 (50.1)	156 (83.9)	30 (16.1)	
BMI (kg/m^2^)				
Mean (SD)	25.05 (3.19)	25.14 (3.25)	24.33 (2.68)	0.124
< 23.0	83 (22.4)	72 (86.7)	11 (13.3)	0.139
23.0–24.9	101 (27.2)	85 (84.2)	16 (15.8)	
≥ 25.0	171 (46.1)	157 (91.8)	14 (8.2)	
Education (years)				
> 12	183 (49.3)	166 (90.7)	17 (9.3)	0.328
12	108 (29.1)	94 (87.0)	14 (13.0)	
< 12	78 (21.0)	66 (84.6)	12 (15.4)	
Unknown	2 (0.5)			
Monthly income (10,000 Won/month)				
≥ 600	114 (30.7)	106 (93.0)	8 (7.0)	0.152
400–599	78 (21.0)	67 (85.9)	11 (14.1)	
200–399	113 (30.5)	100 (88.5)	13 (11.5)	
< 200	54 (14.6)	44 (81.5)	10 (18.5)	
Unknown	12 (3.2)			
Medication				
Hypertension	207 (55.8)	184 (88.9)	23 (11.3)	0.747
Diabetes mellitus	72 (19.4)	67 (93.1)	5 (6.9)	0.219
Hyperlipidemia	153 (41.2)	139 (90.8)	14 (9.2)	0.251

### Comparison of blood pressure variability according to depressive mood

In Table 2, we analyzed the average values of indices representing systolic BPV according to the presence or absence of depressive mood. The values for the intra-individual SD were as follows: total, 10.29 (5.57); no depressive mood, 9.67 (5.66); depressive mood present, 10.16 (4.89). CV exhibited the following values: total, 7.80 (4.00); no depressive mood, 7.76 (4.92); depressive mood present, 8.18 (3.81). Additionally, VIM values were: total, 8.99 (5.52); no depressive mood, 8.94 (5.65); depressive mood present, 9.43 (4.39). Lastly, ARV was recorded as follows: total, 10.89 (7.91); no depressive mood, 10.81 (8.02); depressive mood present, 11.48 (7.11). Considering a mean ARV of 10.89 (7.91) across the entire cohort, high visit-to-visit BPV was defined as an ARV exceeding 10.

**Table 2:** Comparison of systolic blood pressure variability according to depressive mood

Characteristics	Total	Participants without depressive mood	Participants with depressive mood	P-value
	(*n* = 371)	(*n* = 328)	(*n* = 43)	
	Mean (SD)			
**Total Participants**				
Baseline SBP	126.18 (13.20)	126.49 (13.02)	123.77 (14.44)	0.203
Baseline DBP	77.46 (10.06)	77.43(10.05)	77.65 (10.25)	0.894
Intra-individual standard deviation	10.29 (5.57)	9.67 (5.66)	10.16 (4.89)	0.585
Coefficient of variation	7.80(4.0)	7.76(4.92)	8.18(3.81)	0.586
Variation independent of mean	8.99(5.52)	8.94(5.65)	9.43(4.39)	0.585
Average real variability	10.89(7.91)	10.81(8.02)	11.48(7.11)	0.604
**Participants without hypertension **(***N* = 164)**				
Baseline SBP	121.79 (11.93)	122.10(11.65)	119.55(13.91)	0.373
Baseline DBP	74.79 (8.52)	75.12 (8.39)	73.45 (9.24)	0.19
Intra-individual standard deviation	8.63 (4.95)	8.58 (4.93)	8.94 (5.19)	0.763
Coefficient of variation	6.99 (3.76)	6.94 (3.74)	7.28 (4.02)	0.81
Variation independent of mean	8.05 (4.34)	8.00 (4.32)	8.38 (4.63)	0.712
Average real variability	10.15 (8.29)	10.09 (8.27)	10.52 (8.65)	0.832
**Participants with hypertension**				
Baseline SBP	129.00(12.63)	129.33(12.40)	126.40(14.42)	0.294
Baseline DBP	79.19(10.21)	79.02(10.26)	80.59(9.91)	0.489
Intra-individual standard deviation	10.59(5.88)	10.51(6.04)	11.22(4.46)	0.588
Coefficient of variation	8.45(5.41)	8.39(5.61)	8.96(3.52)	0.632
Variation independent of mean	9.74(6.21)	9.67(6.43)	10.33(4.06)	0.631
Average real variability	11.48(7.57)	11.38(7.80)	12.32(5.51)	0.575

### Logistic regression analysis of associations of high visit-to-visit BPV with participant characteristics and depressive mood

Table 3 presents the individual ORs for factors associated with high visit-to visit BPV. We estimated univariate ORs for age, sex, BMI, education, income, use of antihypertensive medication, and depressive mood. Factors associated with high visit-to-visit blood pressure variability (BPV) include age of 70 years or older (OR: 4.43, 95% CI: 1.63–12.04, *P* = 0.004), an education lower than high school level (OR: 1.82, 95% CI: 1.00–3.30, *P* = 0.049), and use of antihypertensive medication (OR: 1.55, 95% CI: 1.02–2.35, *P* = 0.040).

**Table 3:** Comparison of characteristics according high systolic BPV based on ARV of systolic blood pressure

	High BPV		Univariate OR (95% CI)	*P*-value
	*N*(%)			
	No	Yes		
Age (years)				
< 50	24(70.59)	10(29.41)	1(Reference)	
50–59	66(54.10)	56(45.90)	2.04(0.90–4.62)	0.089
60–69	108(60.67)	70(39.33)	1.56(0.70–3.45)	0.277
≥ 70	13(35.14)	24(64.86)	4.43(1.63–12.04)	0.004
Sex				
Men	103(55.68)	82(44.32)	1(Reference)	
Women	108(58.06)	78(41.94)	0.91(0.60–1.37)	0.642
BMI (kg/m^2^)				
< 23.0	49(59.04)	34(40.96)	1(Reference)	
23.0–24.9	61(60.40)	40(39.60)	0.95(0.52–1.71)	0.852
≥ 25.0	91(53.22)	80(46.78))	1.27(0.75–2.15)	0.382
Education (years)				
> 12	44(67.69)	21(32.31)	1(Reference)	
12	67(55.37)	54(44.63)	1.69(0.90–3.18)	0.104
< 12	98(53.55)	85(46.45)	1.82(1.00-3.30)	0.049
Income (10,000 won/month)				
≥ 600	32(59.26)	22(40.74)	1(Reference)	
400–599	76(67.26)	37(32.74)	0.71(0.36–1.38)	0.313
200–399	40(51.28)	38(48.72)	1.38(0.69–2.79)	0.366
< 200	55(48.25)	59(51.75)	1.56(0.81–3.01)	0.183
Antihypertensive medication				
No	103(62.80)	61(37.20)	1(Reference)	
Yes	108(52.17)	99(47.83)	1.55(1.02–2.35)	0.040
Depressive mood				
No	190(57.93)	138(42.07)	1(Reference)	
Yes	21(48.84)	22(51.16)	1.44(0.76–2.73)	0.260

In the multivariable analysis, the entire cohort was stratified into two groups: those prescribed antihypertensive medication and those who were not (Table 4). The influence of each factor on high visit-to-visit BPV was then evaluated. Significant associations were observed when stratified by age 70 years or older (OR 7.32, 95% CI: 2.40–21.83). Additionally, associations were found in the non-antihypertensive medication groups (OR 11.63, 95% CI: 1.73–78.23) and antihypertensive medication groups (OR: 9.80, 95% CI: 0.76–125.89). Furthermore, when monthly income was less than 2 million won, the association was observed in the antihypertensive medication groups (OR: 3.10, 95% CI: 1.09–8.82), as well as when exhibiting depressive symptoms in the antihypertensive medication groups (OR: 2.95, 95% CI: 1.06–8.20). Conversely, depressive mood was not associated with the absence of antihypertensive medication use.

**Table 4:** Multivariate logistic regression analysis of factors associated with high SBP variability

	Total		Without HTN medication		With HTN medication	
	OR (95% CI)	*P*-value	OR (95% CI)	*P*-value	OR (95% CI)	*P*-value
Age (years)						
	1.04 (1.01–1.07)	0.006	1.03 (0.99–1.08)	0.123	1.05 (1.00-1.11)	0.032
Sex						
Men	1(Reference)		1(Reference)		1(Reference)	
Women	1.17(0.72–1.91)	0.522	0.80(0.36–1.74)	0.567	1.50(0.77–2.93)	0.237
BMI (kg/m^2^)						
< 23.0	1(Reference)		1(Reference)		1(Reference)	
23.0–24.9	1.04(0.55–1.97)	0.897	1.75(0.72–4.28)	0.218	0.61(0.22–1.65)	0.329
≥ 25.0	1.57(0.87–2.84)	0.133	1.05(0.41–2.68)	0.925	1.45(0.60–3.52)	0.409
Education (years)						
> 12	1(Reference)		1(Reference)		1(Reference)	
12	1.47(0.71–3.03)	0.302	1.08(0.31–3.75)	0.899	1.58(0.60–4.17)	0.356
< 12	1.77(0.85–3.69)	0.125	1.37(0.40–4.63)	0.614	1.87(0.70–5.04)	0.214
Income (10,000 won/month)						
≥ 600	1(Reference)		1(Reference)		1(Reference)	
400–599	0.66(0.31–1.42)	0.289	0.24(0.06–0.96)	0.044	1.18(0.45–3.12)	0.731
200–399	1.29(0.57–2.91)	0.540	0.90(0.22–3.75)	0.889	1.73(0.60–4.98)	0.308
< 200	1.82(0.82–4.03)	0.139	1.20(0.30–4.76)	0.798	3.10(1.09–8.82)	0.034
Depressive mood						
No	1(Reference)		1(Reference)		1(Reference)	
Yes	1.56(0.77–3.19)	0.220	0.71(0.22–2.32)	0.575	2.95(1.06–8.20)	0.038

## Discussion

In this study, we observed a three-fold higher OR for high visit-to-visit BPV in patients taking antihypertensive medication when they had depressive mood. Additionally, we identified older age as a factor associated with high SBP variability. These findings suggest the importance of monitoring BPV in patients visiting primary care, particularly those showing symptoms of depressive mood or those with older age, especially among individuals taking antihypertensive medication.

A previous study demonstrated that elevated visit-to-visit BPV increases the risk of cardiovascular disease and is a significant predictor of cardiovascular outcomes [22]. Higher systolic BPV was associated with a higher incidence of cardiovascular events and mortality [23]. Additionally, another study indicated that the multivariable-adjusted HRs and 95% CIs for the quartiles of the SD of systolic BPV, compared with the first quartile, were incrementally higher for quartiles 2 through 4, demonstrating a progressive increase in risk [13]. Thus, monitoring BPV is crucial for assessing the risk of cardiovascular disease among patients visiting primary care.

Several studies have reported autonomic dysfunction in individuals with depression [24–26], characterized by elevated plasma or urinary levels of catecholamine compared with controls [24]. Additionally, individuals with depression may exhibit heightened heart rate responses to physical or psychological stressors, even in the absence of other medical conditions [24, 26]. Building upon these findings, the present study suggests that depressive mood can impact BPV, potentially due to autonomic dysfunction in patients with depressive mood. In line with our research, a recent study indicated an association between depression and diastolic BPV [27]. However, it’s important to note that the individuals in this study were derived from the Alzheimer’s Disease Neuroimaging Initiative database. Similar to our findings, another study demonstrated elevated systolic BPV among adolescents with major depression [28], attributing it to an overactivity of the cardiovascular sympathetic nervous system. Furthermore, our results align with the findings from a separate study indicating an association between increased systolic BPV and the prevalence of late-onset depression [17].

Our findings revealed that older age was associated with high SBP variability, aligning with prior research that has consistently reported an association between BPV and advanced age [29–32]. This association could be attributed to the impact of increased arterial stiffness in older age, leading to alterations in the arterial vessel wall and subsequently contributing to increased BPV [11]. Notably, our study population comprised primary care patients with and without hypertension. Interestingly, we observed that participants taking antihypertensive medication were more likely to exhibit SBP variability than those who were not.

The impact of antihypertensive medication on BPV can vary based on the specific class of medication [33]. A meta-analysis has suggested that calcium channel blockers may decrease long-term BPV, while angiotensin receptor blockers, ACE inhibitors, and beta-blockers could be associated with an increase in BPV [34]. In our study, we categorized patients into two groups according to the use of antihypertensive medication, without specifying the type of medication. Therefore, future investigations may be warranted to consider the effects of specific classes of antihypertensive medication on BPV. Furthermore, SBP variability has been linked to mortality [30, 35, 36] and cardiovascular diseases [35, 37, 38]. Therefore, SBP variability may serve as a valuable indicator of variations in morbidity and mortality compared with DBP variability.

This study has several limitations. First, BP was solely measured during clinic visits, without incorporating at-home BP measurements or daily BPV. Second, the assessment of depressive mood was conducted only once, at study recruitment, precluding the evaluation of mood changes over time and potential associations with changes in BPV during follow-up visits. The lack of data on evolving mood status limits the ability to establish a temporal relationship between mood fluctuations and BPV. Additionally, there is a potential for selection bias, given that only 40% of initial participants attended follow-up visits. This could be influenced by factors such as strong doctor–patient relationships and high adherence among those who attended follow-up appointments. However, it’s important to note that this may not affect the association between depressive mood and BPV, given that BPV may not be directly associated with patient adherence. Finally, despite adjusting for several potential confounding factors such as age, sex, BMI, and socioeconomic status, the presence of unmeasured residual confounding factors cannot be ruled out. Furthermore, the inclusion of patients with chronic diseases such as diabetes [39, 40] in the study population may introduce additional factors that may have affected their BPV.

Despite these limitations, our study is meaningful because it examined BPV among primary care patients, utilizing a standardized questionnaire (CES-D scale) to assess depressive mood [20]. The findings of our study underscore the importance of closely monitoring BPV in patients with depressive mood, older age, and those prescribed antihypertensive medication. Notably, for primary care clinics treating patients with depressive mood, vigilant monitoring of visit-to-visit SBP may be crucial for optimizing patient outcomes. Additionally, although our study helps elucidate the association between depressive mood and BPV, we could not evaluate whether the improvement of depressive mood could decrease BPV in primary care patients. Further study is warranted to explore this association in a larger number of participants with extended follow-ups to offer greater insights.

## Conclusion

Based on our findings, the close monitoring of BPV in patients with depression among patients taking antihypertensive medication is crucial for optimizing treatment outcomes. As symptoms of depression are commonly encountered in clinical practice, our study highlights the necessity for a comprehensive approach to manage not only depression but to monitor BPV in these patient populations.

## Data Availability

The data are not publicly shared because we do not have permission from the Institutional Review Board to distribute the data. The analytic methods are available from the corresponding authors upon reasonable request.

## Abbreviations

BMI: Body mass index
BP: Blood pressure
BPV: Blood pressure variability
CES-D: Center for Epidemiologic Studies Depression
CI: Confidence interval
DBP: Diastolic blood pressure
FACTS: Family Cohort Study in Primary Care
OR: Odds ratio
SBP: Systolic blood pressure
SD: Standard deviation
